# COVID-19 and Artificial Intelligence: An Approach to Forecast the Severity of Diagnosis

**DOI:** 10.3390/life11111281

**Published:** 2021-11-22

**Authors:** Anca Loredana Udriștoiu, Alice Elena Ghenea, Ștefan Udriștoiu, Manuela Neaga, Ovidiu Mircea Zlatian, Corina Maria Vasile, Mihaela Popescu, Eugen Nicolae Țieranu, Alex-Ioan Salan, Adina Andreea Turcu, Dragos Nicolosu, Daniela Calina, Ramona Cioboata

**Affiliations:** 1 Faculty of Automation, Computers and Electronics, University of Craiova, 200776 Craiova, Romania; anca.udristoiu@edu.ucv.ro (A.L.U.); stefan.udristoiu@edu.ucv.ro (Ș.U.); neagamanuela@yahoo.com (M.N.); 2 Department of Bacteriology-Virology-Parasitology, University of Medicine and Pharmacy of Craiova, 200349 Craiova, Romania; ovidiu.zlatian@gmail.com; 3 PhD School Department, University of Medicine and Pharmacy of Craiova, 200349 Craiova, Romania; corina.vasile93@gmail.com; 4 Department of Endocrinology, University of Medicine and Pharmacy of Craiova, 200349 Craiova, Romania; mihaela.n.popescu99@gmail.com; 5 Department of Cardiology, University of Medicine and Pharmacy of Craiova, 200642 Craiova, Romania; tieranueugen@gmail.com; 6 Department of Oral and Maxillofacial Surgery, University of Medicine and Pharmacy Craiova, 200349 Craiova, Romania; alex.salan@umfcv.ro; 7 Infectious Disease Department, Victor Babes University Hospital Craiova, 200515 Craiova, Romania; adinaturcu14@yahoo.com; 8 Pneumology Department, Victor Babes University Hospital Craiova, 200515 Craiova, Romania; nicolosud@yahoo.com; 9 Department of Clinical Pharmacy, University of Pharmacy and Medicine Craiova, 200349 Craiova, Romania; 10 Department of Pneumology, University of Pharmacy and Medicine Craiova, 200349 Craiova, Romania; ramona.cioboata@umfcv.ro

**Keywords:** COVID-19, artificial intelligence, deep learning

## Abstract

(1) Background: The new SARS-COV-2 pandemic overwhelmed intensive care units, clinicians, and radiologists, so the development of methods to forecast the diagnosis’ severity became a necessity and a helpful tool. (2) Methods: In this paper, we proposed an artificial intelligence-based multimodal approach to forecast the future diagnosis’ severity of patients with laboratory-confirmed cases of SARS-CoV-2 infection. At hospital admission, we collected 46 clinical and biological variables with chest X-ray scans from 475 COVID-19 positively tested patients. An ensemble of machine learning algorithms (AI-Score) was developed to predict the future severity score as mild, moderate, and severe for COVID-19-infected patients. Additionally, a deep learning module (CXR-Score) was developed to automatically classify the chest X-ray images and integrate them into AI-Score. (3) Results: The AI-Score predicted the COVID-19 diagnosis’ severity on the testing/control dataset (95 patients) with an average accuracy of 98.59%, average specificity of 98.97%, and average sensitivity of 97.93%. The CXR-Score module graded the severity of chest X-ray images with an average accuracy of 99.08% on the testing/control dataset (95 chest X-ray images). (4) Conclusions: Our study demonstrated that the deep learning methods based on the integration of clinical and biological data with chest X-ray images accurately predicted the COVID-19 severity score of positive-tested patients.

## 1. Introduction

The emergence and extremely rapid spread of the new coronavirus, COVID-19, has led to the existence of a global problem, a problem never seen before. International organizations have come to the aid of the states of the world and have proposed numerous legislative instruments for combating and spreading this virus. The pandemic context made its presence felt at a rather difficult time nationally and globally [1]. This global challenge, SARS-CoV-2, overcomes several intensely debated issues, such as respect for human rights and freedoms, environmental protection, respect for democracy, and the reduction of social and economic imbalances that can influence the spread of the disease [2].

Researchers have tried developing more effective prediction models to control the spread of COVID-19 and discovered new, unexpected factors that can influence the severity of the disease [3,4]. With the appearance of COVID-19, more attention has been paid to improve automatic recognition systems based on artificial intelligence methods. It is difficult to provide an easy solution to this problem. However, precise and effective artificial intelligence techniques can be useful in overcoming this pandemic and providing adequate care to patients.

Forecasting the severity of COVID-19 patients is a crucial step in patients’ management since the patients’ treatment depends on it: The mild and moderate COVID−19 cases necessitate antivirals and oxygen therapy, while severe ones necessitate intensive care units or ventilator support [5].

Many studies showed how important is to anticipate the COVID-19 severity since the evolution to critical disease stage could be quick, due especially to the immune mechanisms [6,7]. Systems based on deep learning algorithms were developed to diagnose COVID-19 disease, using different medical imaging modalities like Computer Tomography (CT) and X-ray [8].

In [9,10], chest X-ray images were investigated to differentiate lung changes produced by COVID-19 disease. These studies demonstrated that the prediction of COVID-19 disease severity could be also established based on lung changes as ground-glass opacity, lungs’ involvement, consolidation, bilateral infiltration, and vascular enlargement.

Together with the patterns of lung CT or X-ray images, other parameters taken into consideration for predicting the severity were symptoms and clinical and biological tests of COVID-19-infected patients [11].

In [12,13,14], the authors investigated different machine learning algorithms to investigate the correlations between usual blood routines and COVID-19 diagnosis. In [12], an ensemble of four machine learning algorithms (support vector machines, adaptive boosting, random forest, and k-nearest neighbours) was developed to investigate if normal, usual blood medical tests can be used to detect COVID-19 infection. The conclusion of the study [12] was that only the usual blood tests did not help in accurate detection of COVID-19.

In [15], a screening method based on machine-learning was proposed to predict a positive SARS-CoV-2 infection, taking into consideration eight symptoms and features, such as sex, age, cough, fever, sore throat, shortness of breath, headache, and known contact with a person confirmed to have COVID-19.

The scope of the study proposed in [16] was to develop an artificial intelligence algorithm capable of correlating possible findings on chest CT of patients, symptoms of respiratory syndromes, and positive epidemiological factors with the evolution of the COVID-19 disease.

In our study, we designed a multimodal approach for predicting the future severity of diagnosis of COVID-19-infected patients at early disease stages. The proposed method integrated information from multiple sources (chest X-ray images, symptoms, clinical and biological variables), using an ensemble of deep learning (DL), pre-trained models and ensemble with stacking of machine learning (ML) algorithms.

For classifying chest X-ray images (CXR), we developed an ensemble (CXR-Score) composed of four pre-trained models: VGG-19, ResNet50, DenseNet121, and Inception v3. These DL models were trained on the ImageNet dataset, being capable of recognizing 1000 object classes. Using transfer learning with fine-tuning, they can be used in diverse domains. The method consists of removing the final set of fully connected layers of the pre-trained network and replacing them with a new set of fully connected layers with random initializations [17,18].

For classifying the patients’ symptoms and biological and clinical data, we developed an ensemble called AI-Score based on stacking. This module combined four base models (Ada Boost, Random Forest, and XGBoost) and their configurations to make better predictions than a single model [19].

The ensemble-based method was successfully applied to improve the accuracy of individual DL models and ML algorithms, even if we had a small patient dataset.

## 2. Materials and Methods

### 2.1. Multimodal Approach Description

**Figure 1:**
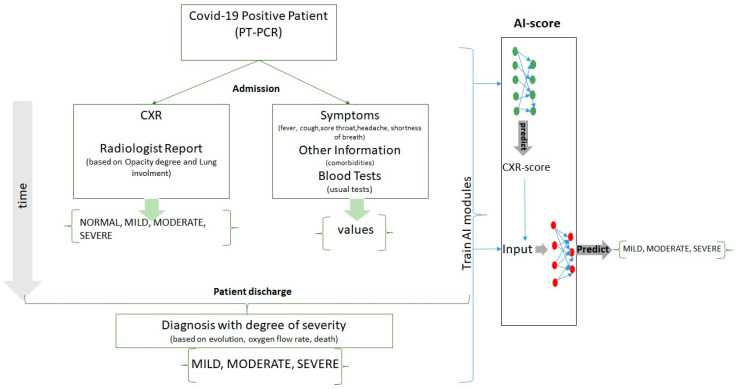
The pipeline of the proposed method for forecasting the COVID-19 severity score.

Our multimodal approach consisted of the following steps, described in Figure 1.

1. At admission, from positive-tested COVID-19 patients, we collected the symptoms, clinical variables, blood tests, and chest X-ray scans together with a radiologist’ report.
2. During hospitalization, each patient was diagnosed with a COVID-19 severity score (mild, moderate, and severe) assessed by the oxygen flow rate, the necessity of mechanical ventilation, or patient death.
3. We constructed modules based on artificial intelligence that were trained on data collected from patients with COVID-19 and could predict the future severity of the diagnosis.

### 2.2. Retrospective Study

The retrospective study included 475 patients with laboratory-confirmed cases of SARS-CoV-2 infection, admitted between September 2020 and May 2021 to Victor Babes University Hospital (Craiova, Romania), the only COVID Hospital in the Oltenia Region. Patients were included in the study if they were admitted directly into one of the two Infectious Disease Clinics or into one of the two Pneumology Departments. Patients confirmed with SARS-CoV-2 infection and transferred from other non-profile hospitals in the region were also included.

Patients who did not undergo laboratory confirmation of SARS-CoV-2 or who tested negative in the laboratory of Victor Babes University Hospital or Human Genomics Laboratory as part of the Craiova Center of Diagnostic and Treatment were excluded from this research.

Epidemiological and clinical data were analyzed among those with abnormal or normal imaging findings. The chest X-ray scans were performed using three different radiography systems, one of which was mobile and two of which were fixed.

This study was carried out following the Helsinki Declaration of 1975 and was approved by the Review Ethics Board of the Victor Babes University Hospital.

### 2.3. Clinical and Biological Variables

We collected data from electronic patient records, using custom queries to extract the values from the database. All values were measured at admission to the hospital. These input parameters were age, sex, respiratory parameters (oxygen saturation, respiratory rate), cardiovascular parameters (systolic pressure, diastolic pressure, cardiac frequency), body temperature, symptoms (coughing, sore throat, headache, shortness of breath, vertigo, palpitations, physical asthenia, abdominal pain, myalgia, inappetence, diarrhoea), associated comorbidities (diabetes, cardiac disease, kidney disease, asthma, hypertension, autoimmune thyroid, obesity), full blood count, biochemical parameters (aspartate aminotransferase (AST), alanine aminotransferase (ALT), glucose, total bilirubin, creatinine, urea), inflammatory parameters (erythrocyte sedimentation rate (ESR), C reactive protein (CRP), fibrinogen), and D-dimers.

To assess the severity of diagnosis we used chest X-ray images, parameters of respiratory function (oxygen saturation), and hematological parameters based on World Health Organization (WHO) COVID-19 disease severity: mild disease (symptomatic patients confirmed with SARS-CoV-2 infection but without signs of viral pneumonia or hypoxia), moderate disease (patients with clinical signs of pneumonia but on the chest X-ray no signs of severe pneumonia and SpO2 ≥ 90% on room air), severe disease (patients with clinical and radiological signs of severe pneumonia plus SpO2 < 90% on room air or respiratory rate >30 breaths/min) [20].

**Figure 2:**
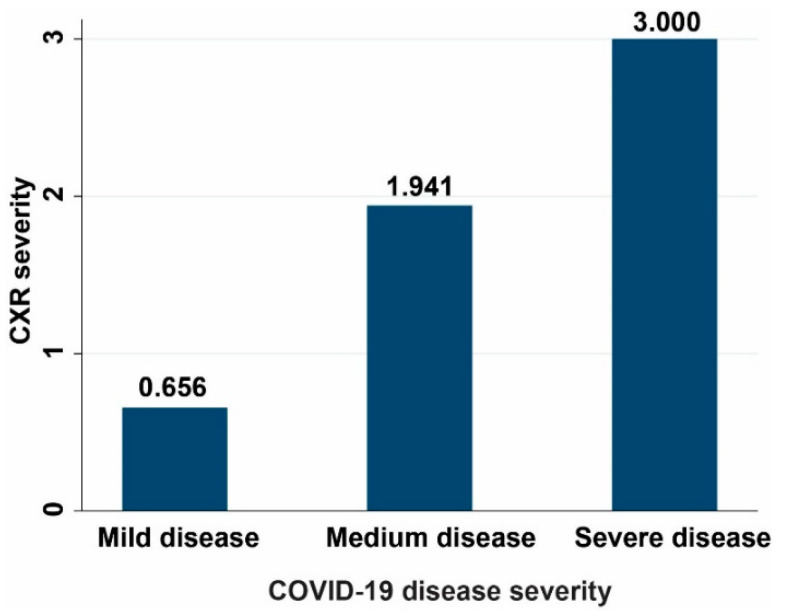
The correlation between chest X-ray and COVID-19 severity.

The correlation between chest X-ray and COVID-19 severity in our patients’ cohort can be visualized in Figure 2:

### 2.4. Chest X-ray Image Acquisition and Radiologist Report

Many current types of research established that opacity and lung involvement were important guidelines for the future evolution of COVID-19 disease [21,22].

The Italian Society of Radiology (SIRM) strongly recommends using chest X-ray as a first-line imaging tool and reserved CT examination for more severe cases [23,24,25]. In our center, the CT scan is usually performed after the chest X-ray and only in specific situations.

In our study, the chest X-ray images were collected from patients who were positive tested for COVID-19. The X-ray images were labelled by radiologists with more than 10 years of experience. The severity score of lung illness was based on opacity degree and the lung involvement established from chest X-ray images of the COVID-19 patients.

The CXR severity score was adapted from Irmak [9] and Wong et al. [26], and it was between 0 and 14 by summing up the opacity (0–6) and involvement (0–8). The total severity score summed the individual scores of both lungs.

Therefore, the chest X-ray images used for training our models were manually classified by the radiologists as normal (without modifications, with a total severity score of 0), mild (total severity score of 1–6), moderate (total severity score of 7–12), or severe (total severity score of >12).

**Figure 3:**
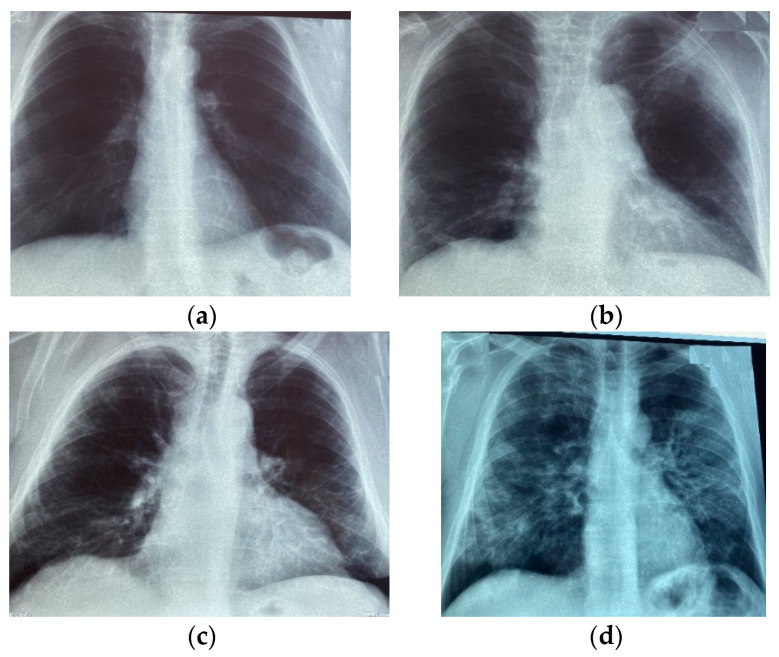
Chest X-ray images labelled with different COVID-19 severity score: (**a**) Normal; (**b**) Mild; (**c**) Moderate; (**d**) Severe.

In Figure 3, chest X-ray images with different severity scores can be observed.

### 2.5. Datasets

We used two datasets (CXR-Dataset and SCB-Dataset) for training and testing the proposed method.

From 475 patients, 380 patients were used for training and validation, while 95 control patients were used for testing.

The image dataset (CXR) contained the chest X-ray images of our patients’ cohort (475); the images were labelled by a radiologist with four severity grades (normal, mild, moderate, severe). The initial chest X-ray image dataset was split into training (380) and testing (95) datasets. Only the training dataset was augmented to obtain the desired invariance and robustness of algorithms, using the following methods: brightness changes, contrast adjustment, and parallel shifting. The augmented training dataset (CXR-Dataset) contained 2092 images, which were used to learn (80% images) and validate (20% images) the algorithms. The testing dataset contained the images of those 95 control patients and was used only for testing the ability of DL models.

The second dataset consisted of patients’ symptoms and clinical and biological variables collected at patients’ admission and labelled with the diagnosis (mild, moderate, or severe) established at patients’ discharge. This initial dataset was split into two independent patients’ datasets: training (380) and testing (95). In the training dataset, together with symptoms and clinical and biological variables, the radiologic lung degree of severity was taken into consideration, while in the testing dataset we used the deep learning CXR-Score. The SCB training dataset was used for learning and validation of the proposed ML methods, while the SCB testing dataset was used only for testing the methods. The transformations applied on the SCB Dataset were binarization and normalization transformations, while the problem of missing values was resolved by replacing them with the mean values.

**Figure 4:**
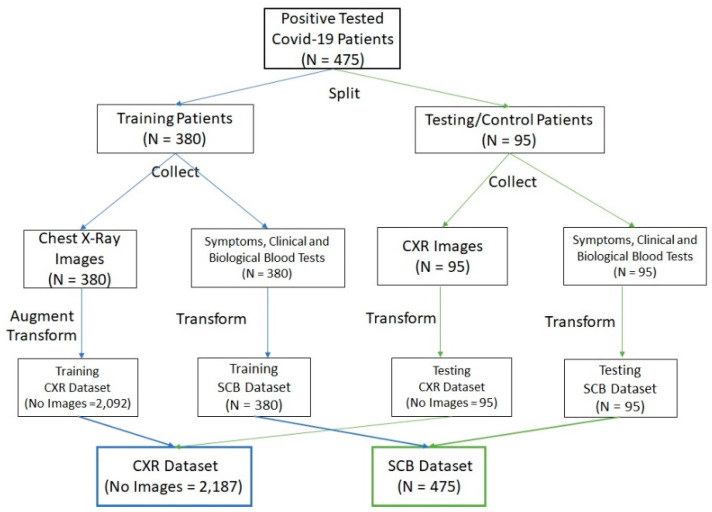
The description of constructing the CXR and SCB datasets.

The datasets were constructed as in Figure 4.

Additionally, the distribution of patients between diagnoses can be observed in Table 1.

### 2.6. CXR-Score Module

This module was able to automatically classify the CXR images with four severity scores: mild, moderate, severe, and normal.

We proposed an ensemble that fused four pre-trained models (VGG, ResNet, DenseNet, and Inception) fine-tuned with our train dataset. The performance of classifying the severity of CXR images was improved using an ensemble method that reduced the variance by training four models instead of a single model and by combining the predictions of the models.

**Figure 5:**
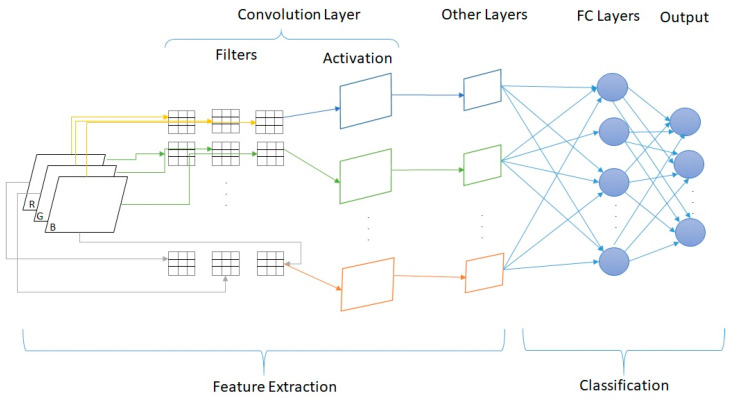
Generic architecture of a DL convolutional neural network.

A generic architecture of a DL convolutional neural architecture can be observed in Figure 5. The convolutional layers were interposed with pooling and batch normalization layers for the feature extraction task, whereas the fully connected (FC) layers were used for the classification task [18].

#### 2.6.1. VGG Model

The VGG architecture was proposed by Simonyan et al. [27], having a depth between 16 and 19 layers and consisting of very small convolution filters. For our proposed VGG model with transfer learning, we used the configuration consisting of 19 convolutional layers, with filters of size 3 × 3.

The proposed VGG-19 model is described in Algorithm 1.
**Algorithm 1.** The VGG-19 model description.Input: CXR images of dimension 500 × 500 pixels from the Training CXR Dataset Output: VGG model weights 1. epochs ← 100 2. for each image in the dataset do 3.  resize the image to 224 × 224 pixels 4.  normalize the image pixels values from (0,255) to (0,1) 5. end 6. Load the VGG-19 model pre-trained on the ImageNet dataset 7. Remove the last layer of the model 8. Make non-trainable all the layers of the model 9. Add a Flatten layer to the model output to obtain 1-D array of features 10. Apply a batch normalization to the 1-D array of features 11. Add a fully connected layer with 256 hidden neurons 12. Apply a dropout for inactivate units (40%) in the previous layer 13. Add a fully connected layer with 128 hidden neurons 14. Apply a dropout for inactivate units (60%) in the previous layer 15. Apply a batch normalization 16. Add a fully connected layer with four hidden units and a softmax activation function. 17. Optimize the model with Adam with learning_rate = 0.01 and a decay = learning_rate/epochs 18. Train the model for the given number of epochs and a batch size of 32 19. Save the final model

#### 2.6.2. ResNet Model

What makes convolutional neural networks (CNNs) very effective is their hierarchical structure capable of recognizing visual patterns and features. Therefore, He et al. proposed the ResNet [28] and reformulated the layers as learning residual functions. By comparison to VGG, ResNet is eight times deeper, but still has a lower complexity. In our implementation, we used the ResNet50 version, which has 50 blocks, and each convolutional block has three convolutional layers. The proposed ResNet50 model is described in Algorithm 2.
**Algorithm 2.** The ResNet50 model description.Input: CXR images of dimension 500 × 500 pixels from the Training CXR-Dataset Output: ResNet model weights 1. epochs ← 100 2. for each image in the dataset do 3. resize image to 224 × 224 pixels 4. normalize the image pixels values from (0,255) to (0,1) 5. end 6. Load the ResNet50 model pre-trained on the ImageNet dataset 7. Make non-trainable all the layers of the model 8. Add a Flatten layer to the model output to obtain 1-D array of features 9. Apply a batch normalization to the 1-D array of features 10. Add a fully connected layer with 256 hidden neurons 11. Apply a dropout for inactivate units (50%) in the previous layer 12. Add a fully connected layer with 128 hidden neurons 13. Apply a dropout for inactivate units (50%) in the previous layer 14. Apply a batch normalization 15. Add a fully connected layer with four hidden units and a softmax activation function. 16. Optimize the model with Adam optimizer using a learning_rate = 0.0001 and a decay = learning_rate/epochs 17. Train the model for the given number of epochs and a batch size of 32 18. Save the final model

#### 2.6.3. Inception Model

In 2015, Szegedi et al. in [29] proposed the GoogLeNet consisting of 22 convolutional layers including nine Inception modules. An Inception module has three types of kernel filters 5 × 5, 3 × 3, and 1 × 1 for convolution and a 3 × 3 filter for pooling. GoogLeNet uses stochastic gradient descent (SGD) algorithms during the training stage to extract higher-level features. For our proposed Inception model, we used the version InceptionV3. The proposed InceptionV3 model is described in Algorithm 3.
**Algorithm 3.** The InceptionV3 model description.Input: CXR images of dimension 500 × 500 pixels from the Training CXR-Dataset Output: Inception model weights 1. epochs ← 100 2. for each image in the dataset do 3. resize the image to 224 × 224 pixels 4. normalize the image pixels values from (0,255) to (0,1) 5. end 6. Load the InceptionV3 model pre-trained on the ImageNet dataset 7. Make non-trainable all the layers of the model 8. Add a Flatten layer to the model output to obtain a 1-D array of features 9. Apply a batch normalization to the 1-D array of features 10. Add a fully connected layer with 256 hidden neurons 11. Apply a dropout for inactivate units (40%) in the previous layer 12. Add a fully connected layer with 128 hidden neurons 13. Apply a dropout for inactivate units (60%) in the previous layer 14. Add a fully connected layer with 4 hidden units and a softmax activation function. 15. Optimize the model with RMSprop optimizer using a learning_rate = 0.001 and a decay = learning_rate/epochs 16. Train the model for the given number of epochs and a batch size of 32 17. Save the final model

#### 2.6.4. DenseNet Model

DenseNet was proposed by Huang G. and is an extension of ResNet architecture [30]. A DenseNet is a type of convolutional neural network that utilises dense connections between layers, connecting all layers with matching feature-map sizes directly with each other. Each layer obtains additional inputs from all preceding layers and passes on its feature maps to all subsequent layers.

The proposed DenseNet121 model is described in Algorithm 4.
**Algorithm 4.** The DenseNet121 model description.Input: CXR images of dimension 500 × 500 pixels from the Training CXR-Dataset Output: DenseNet model weights 1. epochs ← 100 2. for each image in the dataset do 3. resize the image to 224 × 224 pixels 4. normalize the image pixels values from (0,255) to (0,1) 5. end 6. Load the DenseNet121 model pre-trained on the ImageNet dataset 7. Make non-trainable all the layers of the model 8. Add a Flatten layer to the model output to obtain a 1-D array of features 9. Apply a batch normalization to the 1-D array of features 10. Add a fully connected layer with 512 hidden neurons 11. Apply a dropout for inactivate units (20%) in the previous layer 12. Add a fully connected layer with 256 hidden neurons 13. Apply a dropout for inactivate units (65%) in the previous layer 14. Apply a batch normalization 15. Add a fully connected layer with four hidden units and a softmax activation function. 16. Optimize the model with Adam optimizer using a learning_rate = 0.001 and a decay = learning_rate/epochs 17. Train the model for the given number of epochs and a batch size of 32 18. Save the final model

#### 2.6.5. CXR-Score Ensemble Model

To improve the accuracy of chest X-ray images, we developed an automated method considering the ensemble of the previously described deep convolutional neural networks.

The probabilities of the four trained models (VGG-19, ResNet50, InceptionV3, and DenseNet121) were averaged to generate new probabilities P(n) for the final diagnosis decision, as in Equation (1):

(1) $$P\left(n\right)=Average\left(P_{1n}+P_{2n}+P_{3n}+P_{4n}\right)$$

where *n* = 1 … 4 represents the number of diagnostics.

The combined predictions from multiple deep learning architectures could introduce a bias, but also reduced the variance of the ensemble model [31].

The algorithm steps for the proposed ensemble are described in Algorithm 5.
**Algorithm 5.** The CXR-Score ensemble description.Input: CXR images of dimension 500 × 500 pixels from the Testing CXR Dataset Output: prediction probabilities for each diagnosis class (Normal, Mild, Moderate, Severe) 1. for each image in the dataset do 2. resize the image to 224 × 224 pixels 3. normalize the image pixels values from (0,255) to (0,1) 4. end 5. Load the trained VGG-19 model 6. Load the trained InceptionV3 model 7. Load the trained ResNet50 model 8. Load the trained DenseNet121 model 9. Predict the images with VGG-19 resulting in a list of probabilities (P_11_, P_12_, P_13_, P_14_) 10. Predict the images with InceptionV3 resulting in a list of probabilities (P_21_, P_22_, P_23_, P_24_) 11. Predict the images with ResNet50 resulting in a list of probabilities (P_31_, P_32_, P_33_, P_34_) 12. Predict the images with DenseNet121 resulting in a list of probabilities (P_41_, P_42_, P_43_, P_44_) 13. Average the four lists of predictions of the four models. 14. for each class in the set of diagnoses do 15. output prediction probability 16. end

### 2.7. AI-Score Module

To integrate the symptoms, laboratory tests, and chest characteristics of X-ray scans, we developed the machine learning module (AI-score) using a super learner ensemble [16]. The method was capable of detecting and using the interactions among these numerous attributes for our small dataset.

The proposed super learner (AI-score) is an ensemble of machine learning algorithms with two levels. To construct a robust model, we combined in the first level four models that have different prediction methodologies: Ada Boost [32], Random Forests [33], and XGBoost [34]. In the second level, we used the CatBoost algorithm [35].

#### 2.7.1. AdaBoost Model

The AdaBoost algorithm was first introduced by Freund and Schapire in 1996 and belongs in part to the family of boosting algorithms [32]. The algorithm methodology is to sequentially grow decision trees as weak learners. The algorithm is learned from previous mistakes by penalizing incorrectly predicted samples by assigning them a larger weight after each round of prediction.

For parameters’ tuning, in our implementation, we set the maximum depth to 15 and the estimators to 200.

#### 2.7.2. Random Forests Model

The random forests algorithm was developed by Breiman in 2001 and is based on the bagging method [33]. The data are bootstrapped by randomly choosing subsamples for each iteration of growing trees. By comparison to AdaBoost, the growth is realized in parallel for Random forests. The reduction of overfitting is realized by combining many weak learners that underfit because they only use a subset of all training samples.

For parameters’ tuning, in our implementation, we set the maximum depth to 15 and the estimators to 200.

#### 2.7.3. XGBoost Model

XGBoost (eXtreme Gradient Boosting) was introduced by Chen and Guestrin in 2016 and uses the concept of gradient tree boosting [34]. XGBoost has the advantages of increased speed and performance and reduced overfitting by introducing regularization parameters. The algorithm is based on gradient-boosted trees that use regression trees in a sequential learning process as weak learners.

For parameters’ tuning, we set the maximum depth to 15, the estimators to 200, and the learning rate to 0.2.

#### 2.7.4. CatBoost Model

CatBoost is a recently open-sourced machine learning algorithm from Yandex [35]. The algorithm is based on a gradient boosting library and returns very good results with less data, even if the deep learning models necessitate an immense amount of data for learning. The algorithm does not need an extensive hyper-parameter tuning and lowers the chances of overfitting, creating more generalized and robust models.

In our implementation, we tuned the following hyper-parameters: Iterations were 15, depth was 3, and the learning rate was 0.1.

#### 2.7.5. AI-Score Ensemble Model

The AI-score model is an application of stacked generalization with two levels.

- In the first level, the base machine learning models (AdaBoost, RandomForests, XGBoost) used the same 5-fold splits of the training data.
- In the second level, a meta-model (CatBoost) was fit on the out-of-fold predictions from each model of the previous level.

In Algorithm 6, we describe the proposed super learner ensemble AI-score.
**Algorithm 6.** The AI-Score ensemble description.Input: SCB training dataset, SCB testing dataset. Output: prediction probabilities for each diagnosis class (Mild, Moderate, Severe) 1. Select a 5-fold split of the train SCB Dataset. 2. base_models = [“Ada Boost”, “Random Forests”,” XGBoost”] 3. meta_model = “CatBoost” 4. For each model in base_models: 5. Evaluate the model using 5-fold cross-validation. 6. Save all out-of-fold predictions. 7. Fit the model on the full training dataset and save. 8. Fit the meta-model on the out-of-fold predictions from the previous layer. 9. Evaluate the model on the SCB testing dataset. 10. For each class in the set of diagnoses do 11.  output prediction probability

### 2.8. Software and Statistical Tools

For selecting the symptoms and clinical and biological variables relevant for diagnosis severity, we used the existing studies, which aimed to identify the importance of various variables in predicting COVID-19 disease severity. All statistical calculations were performed using the software STATA (StataCorp LLC, USA). We used two approaches. One was to apply the Chi2 test and calculate risk ratio (RR) as effect size (for discrete variables) or Student’s t test and calculate Cohen’s d as effect size (for continuous variables) to measure association with the variable severe disease. A second approach was to use logistic regression with the backward elimination based upon likelihood ratio, which retained in the model several parameters.

The performance metrics used to evaluate the proposed methods were accuracy, sensitivity, specificity, positive predictive value, and negative predictive value.

The area under the receiver operating characteristic curve (AUC-ROC) and Precision/Recall curve were also taken into consideration to evaluate the ability of the method of classification. Additionally, we calculated the optimal thresholds for the ROC curves by seeking the maximum of Geometric mean (G-Mean) scores. For the Precision-Recall curves, we calculated the optimal threshold by seeking the maximum of F-measure (F-Score).

We used the TensorFlow and Keras frameworks [36] to implement the deep learning models and the scikit-learn package [37] to implement the machine learning models and to calculate the performance metrics, while matplotlib [38] was used to plot the ROC and Precision/Recall curves.

## 3. Results

### 3.1. Selection of Patient Variables through Statistics

The severe COVID-19 disease was significantly associated with CXR severity, sex, parameters of respiratory function (oxygen saturation, respiratory rate), cardiovascular function (systolic and diastolic pressure, cardiac frequency), associated disease (diabetes, cardiac disease, kidney disease, hypertension, autoimmune thyroid, and obesity), and presence of symptoms (coughing, headache, shortness of breath, vertigo, palpitations, abdominal pain, myalgia, and inappetence). From the hematological parameters, severe disease was associated with the decrease of white blood cells (WBC), lymphocytes (LYM)%, monocytes (MON)%, eosinophils (EOS)%, and basophiles (BAS)%. Regarding biochemical parameters, the severe disease was significantly associated with ALT and glucose decrease. The inflammatory markers (fibrinogen, CRP, and ESR) were also increased in severe disease. There was significant coagulation in the periphery, as shown by D-dimers’ (products of fibrin degradation) increase. Therefore, from 46 initial variables, only 34 variables were found strongly associated with COVID-19 diagnosis’ severity, as in Table 2.

Additionally, the regression analysis revealed a strong association between oxygen saturation and COVID-19 severity, as in Table 3. Other associations were discovered between the risk of severe COVID-19 disease and various risk factors as female sex, diabetes, obesity (very strong association, Odds Ratio = 166). Severe disease was also associated with an increase in white blood cell numbers.

### 3.2. Interpretability of CXR-Score Module

Each DL model (ResNet50, VGG-19, Inceptionv3, and DenseNet121) was trained on the CXR training dataset and evaluated on the CXR testing dataset. The CXR-Score ensemble model was also evaluated on the CXR testing dataset.

As for performance measures, accuracy, sensitivity, specificity, and positive and negative predictive values were computed, and the quantitative results are summarized in Table 4. By comparison, the best results were obtained using the CXR-Score, with an average accuracy of 99.08%, followed by the DenseNet model with an average accuracy of 99.02%. Additionally, the other three models performed very well for all diagnosis’ classes. The ResNet model perfectly classified the normal diagnosis class.

**Figure 6:**
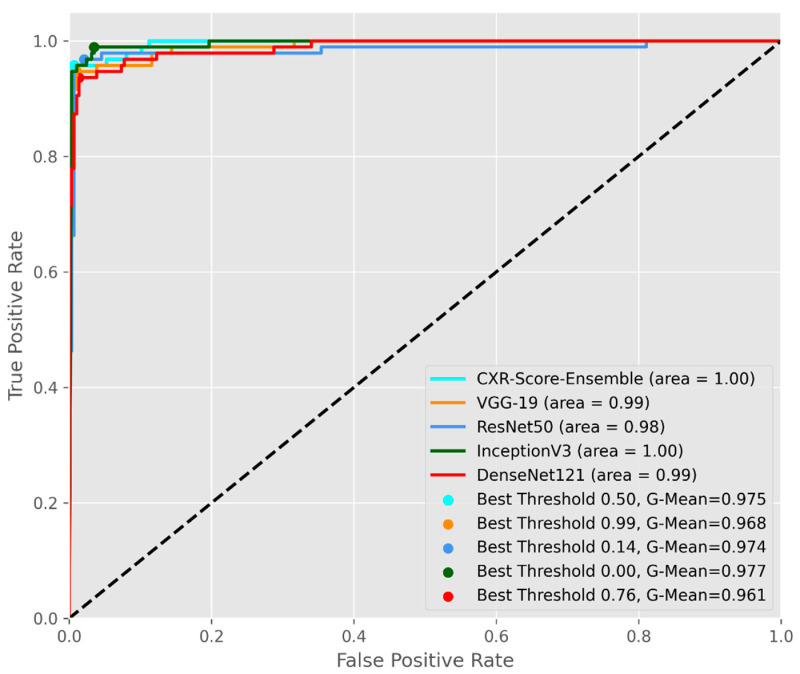
The average AUC-ROC curves for the proposed DL models.

Additionally, each individual DL model and CXR-Score ensemble were evaluated according to the overall scores, computed as the average of the area under the receiver operating characteristic curve (AUC) and the average of G-mean scores, as in Figure 6. CXR-Score and Inception v3 recorded the best G-mean scores and AUCs on the CXR testing dataset.

**Figure 7:**
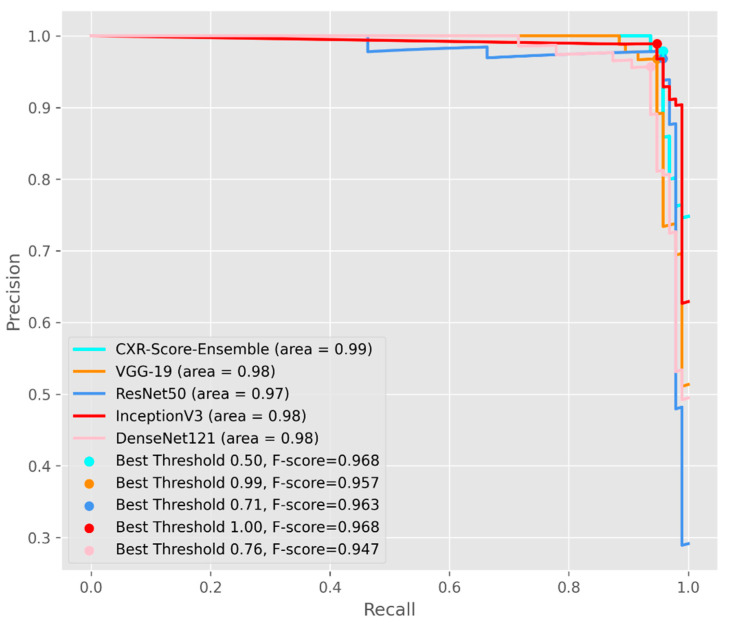
The average Precision/Recall curves for each proposed DL method.

In Figure 7, the Precision/Recall curves together with average precision were evaluated for each model. The CXR-Score ensemble recorded an average precision of 0.99, with the greatest F-measure (F-score) of 0.96 on the CXR testing dataset.

### 3.3. Interpretability of AI-Score Module for Predicting the Final Diagnosis Severity

Each ML model (Random Forests, Ada Boost, XGBoost) was trained on the SCB training dataset and evaluated on the SCB testing dataset. The AI-Score ensemble model was evaluated on the SCB testing dataset. The CXR-Score module was used to predict the chest X-ray severity score for each patient in the SCB testing dataset.

The AI-Score ensemble model recorded the best results for all diagnosis classes, having an average accuracy of 98.59%, average specificity of 98.97%, and average sensitivity of 97.93%. By comparison, the XGBoost algorithm recorded also excellent results, having an average accuracy of 97.89%, average specificity of 98.46%, and average sensitivity of 95.77%. The random forests algorithm obtained an average accuracy of 96.48%, while AdaBoost obtained inferior results for classifying the severe and moderate diagnosis’ classes with an average accuracy of 93.67%. All metrics are summarized in Table 5.

**Figure 8:**
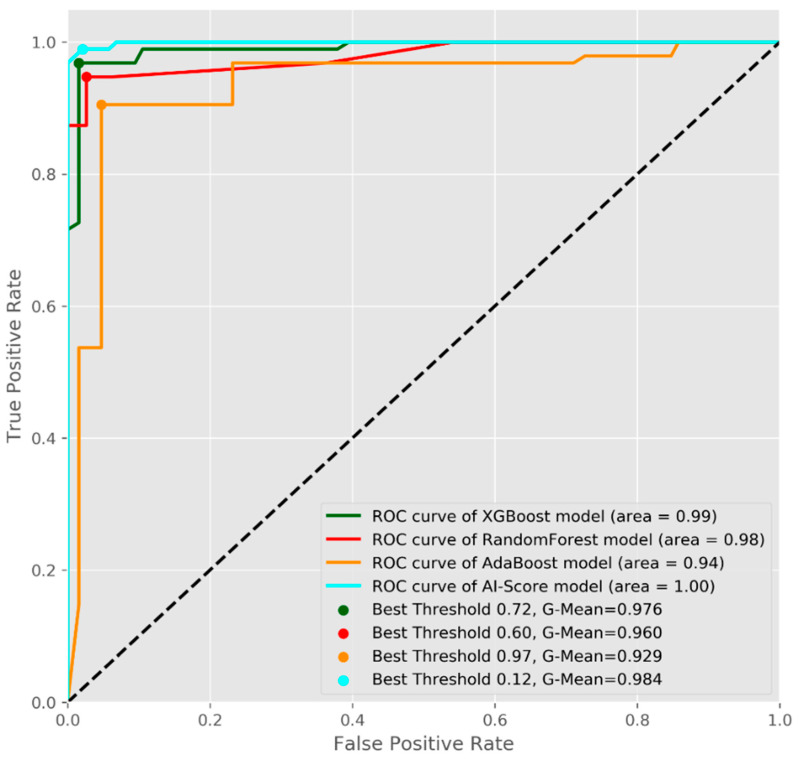
The average AUC-ROC curves for the proposed ML models.

Additionally, each ML model and AI-Score ensemble were evaluated according to the overall scores, computed as the average of the area under the receiver operating characteristic curve (AUC), as in Figure 8. The AI-Score recorded an AUC of 1 and a maximum G-mean of 0.98 for the SCB testing dataset.

## 4. Discussion

Many previous types of research focused on diagnosing patients’ infection with SARS-CoV2 based on chest CT, X-ray scans, symptoms, or blood tests, whereas there were few studies to predict the future COVID-19 severity [10].

In the present study, we developed an artificial intelligence-based method for grading the COVID-19 severity based on multimodal features taken at admission.

We found 34 symptoms and clinical and biological variables that are strongly related to future COVID-19 severity, confirmed also by other studies [39,40,41]. Exploring the clinical features of our patients’ dataset, we found that male and older patients were at risk to develop severe disease. Other clinical variables strongly related to COVID-19 severity were parameters of respiratory function (oxygen saturation, respiratory rate) and cardiovascular function (systolic and diastolic pressure, cardiac frequency). In our study cohort, comorbidities, such as diabetes, cardiac disease, kidney disease, hypertension, autoimmune thyroid, and obesity, were related to the severity and they were also confirmed by studies in [42,43]. Other connections, which we found, were between several symptoms (coughing, headache, shortness of breath, vertigo, palpitations, abdominal pain, myalgia, and inappetence) and COVID-19 severity [44,45].

The hematological parameters, WBC, LYM%, EOS%, and BAS%, increased in severe disease, while MON% and NEU% decreased. PLT (Thrombocytes) were not associated with severity in our study cohort. We also found relationships between D-dimers, fibrinogen, ESR, and severity.

Together with these clinical and biological variables, the chest X-ray images offered important features of disease severity. The lung characteristics that we included were opacity and lung involvement [9,26]. The advantage of the deep learning classification of the chest X-ray images into normal, mild, moderate, and severe was the speed and good classification accuracy, which were even higher than those of a radiologist [10]. Our CXR-Score module had accuracy in identifying the severe and mild pattern in CXR images of 99.54%, while in identifying the moderate pattern in CXR images it was 98.4%. The normal CXR images were classified with an accuracy of 98.85%.

The final prognosis of diagnosis’ severity was established using AI-Score module, in which we integrated 34 variables: symptoms, clinical and biological variables, and CXR-Score. The AI-Score module classified the severe diagnosis with an accuracy of 98.94% and a sensitivity of 97.14%, while the moderate diagnosis was classified with an accuracy of 98.94% and a sensitivity of 96.66%. Regarding the mild diagnosis, the obtained accuracy was 97.89% with a sensitivity of 100%.

Our results suggest that the proposed AI-Score method can become a useful tool in forecasting the future severity of diagnosis at a patient’s admission. Ultimately, this improved diagnosis can be used for assessing the efficacy of vaccination or to evaluate new emerging treatments for COVID-19 [46].

Even if the obtained results were satisfactory, our study had some limitations. Firstly, the machine learning and deep learning methods still need human intervention in the processes of image and data collection.

Second, the dataset known in advance was another limitation because there was a risk of overfitting, even if the training and testing datasets were patient-independent. Future research will evaluate and refine the proposed method on a larger patient database collected from other hospitals.

Third, to improve the resolution quality of CXR images, the wavelet denoising technique and super-resolution deep learning methods will be taken into consideration [47]. Even if they improve the results, they are time consuming in real-time computer-assisted methods, due to computations. So, the trade-off between their complexity and performance in real time will be considered in the future.

Fourth, the severity grading of CXR images could be improved by segmentation to detect more precisely the lung involvement [48].

## 5. Conclusions

The novel COVID-19 has become one of the most acute and severe health problems of the past century. Artificial intelligence-based methods already play an important role in combating the pandemic’s terrible effects.

In this study, we proposed an artificial intelligence-based AI-Score solution, which provides fast and powerful assistance to physicians. We integrated ensemble ML and DL algorithms for forecasting the severity of the COVID-19 diagnosis’ evolution based on 34 input variables. AI-Score consists of an ensemble of machine learning algorithms developed to predict the future severity score as mild, moderate, or severe for COVID-19-infected patients. Additionally, a deep learning module (CXR-Score) was developed to automatically classify the chest X-ray images and integrate them into AI-Score.

Our method achieved good accuracy in retrospective chest X-ray images, symptoms, and clinical and biological blood tests. Based on these promising preliminary results and further testing on larger patients’ cohort, our AI-based method could become an important tool for the computer-aided diagnosis of COVID-19 severity in early stages.

## Figures and Tables

**Table 1:** The distribution of patients in the training and testing/control datasets.

Diagnosis	Training (No Patients)	Testing/Control (No Patients)	Total (No Patients)
Mild	114	30	144
Moderate	127	30	157
Severe	139	35	174
Total	380	95	475

**Table 2:** Our cohort description and association between variables measured at admission and COVID-19 severity diagnosis.

Variable	RR	Difference	Cohen’s d	Statistic	*p* Value
Age ≥ 65 years	1.28	-	-	4.27	0.039 *
Male sex	0.47	-	-	38.04	0.001 *
Oxygen saturation (%)	-	−5.66	−1.29	10.47	0.001 *
Systolic pressure (mm Hg)	-	7.39	0.80	15.1	0.001 *
Diastolic pressure (mm Hg)	-	13.74	1.57	17.51	0.001 *
Respiratory rate (/min)	-	1.18	0.96	9.72	0.001 *
Cardiac frequency (/min)	-	16.15	1.34	12.89	0.001 *
Body temperature (°C)	-	0.14	0.18	1.93	0.054
Coughing	1.57	-	-	12.77	0.001 *
Sore throat	1.19	-	-	1.34	0.246
Headache	1.69	-	-	18.53	0.001 *
Shortness of breath	2.78	-	-	48.75	0.001 *
Vertigo	1.53	-	-	11.34	0.001 *
Palpitations	1.33	-	-	4.13	0.042 *
Physical asthenia	1.34	-	-	2.91	0.088
Abdominal pain	1.35	-	-	4.74	0.029 *
Myalgia	2.83	-	-	72.64	0.001 *
Inappetence	1.35	-	-	5.95	0.015 *
Diarrhea	1.86	-	-	17	0.001 *
Diabetes	1.71	-	-	19.21	0.001 *
Cardiac disease	3.06	-	-	78.46	0.001 *
Kidney disease	1.77	-	-	7.99	0.004 *
Asthma	0.61	-	-	2.96	0.085
Hypertension	4.24	-	-	124.02	0.001 *
Autoimmune thyroid	2.88	-	-	39.3	0.001 *
Obesity	2.53	-	-	56.41	0.001 *
WBC (white blood cells) (×10^3^/mmc)	-	2.60	0.16	1.64	0.010 *
LYM (Lymphocytes) (%)	-	3.43	0.30	2.85	0.005 *
MON (Monocytes) (%)	-	−1.38	−0.48	−5.35	0.001 *
NEU (Neutrophiles) (%)	-	−1.95	−0.17	−1.65	0.100
EOS (eosinophiles) (%)	-	0.31	0.52	5.7	0.001 *
BAS (Basophiles) (%)	-	0.06	0.25	2.67	0.008 *
HGB (Hemoglobin) (g/dL)	-	0.09	0.08	0.78	0.437
PLT (Thrombocytes) (×10^3^/mmc)	-	7568	0.07	0.67	0.500
AST (UI/L)	-	3.13	0.09	1.07	0.285
ALT (UI/L)	-	5.26	0.24	2.3	0.022 *
Glucose (g/dL)	-	33.97	0.27	2.76	0.006 *
ESR (mm/h)	-	13.65	0.52	5.08	0.001 *
Total bilirubin (g/dL)	-	0.003	0.01	0.09	0.924
CRP (mg/L)	-	63.09	3.1	30.26	0.001 *
Creatinine	-	0.07	0.11	1.06	0.289
Urea	-	−2.15	−0.10	−0.93	0.352
Fibrinogen	-	86.83	2.17	24.67	0.001 *
D-Dimers	-	1371	2.37	19.54	0.001 *
CXR Severity	-	1.67	2.89	36.1	0.001 *

* Significant variable.

**Table 3:** Logistic regression analysis of factors associated with disease severity.

Severe Disease	Odds Ratio	Std. Err.	z	*p* > z	[95% Conf. Interval]
Oxygen saturation	0.695636	0.030356	−8.32	<0.001 *	[0.638613, 0.757751]
Sex	0.047451	0.024916	−5.8	<0.001 *	[0.016955, 0.132799]
Age group	1.236799	0.51204	0.51	0.608	[0.549412, 2.784198]
Diabetes	0.052452	0.044864	−3.45	<0.001 *	[0.009811, 0.280428]
Obesity	166.5959	183.348	4.65	<0.001 *	[19.26948, 1440.319]
WBC	1.001285	0.000306	4.2	<0.001 *	[1.000686, 1.001885]
Creatinine	6.825661	10.79885	1.21	0.225	[0.307229, 151.6449]
Urea	1.029222	0.029315	1.01	0.312	[0.973341, 1.088312]
Constant	0.279416	2.470919	−0.14	0.885	[8.3 × 10^−9^, 9409517]

* Significant variable.

**Table 4:** The results of the proposed methods used to classify chest X-ray images.

	ResNet50	VGG-19	Inceptionv3	DenseNet121	CXR-Score
Acc_severe (%)	99	98.85	99.31	99.31	99.54
Se_severe (%)	99.05	97.17	98.11	98.11	98.11
Sp_severe (%)	99.09	99.39	99.69	99.69	100
PPV_severe (%)	97.22	98.09	99.04	99.04	100
NPV_severe (%)	99.69	99.09	99.39	99.39	99.4
Acc_moderate (%)	97.26	97.94	97.71	98.17	98.4
Se_moderate (%)	94.35	94.35	95.16	95.16	96.77
Sp_moderate (%)	98.4	99.36	98.72	99.36	99.04
PPV_moderate (%)	95.9	98.31	96.72	98.33	97.56
NPV_moderate (%)	97.78	97.8	98.10	98.11	98.73
Acc_normal (%)	100	99.54	99.77	99.54	99.54
Se_normal (%)	100	99.08	100	99.08	99.08
Sp_normal (%)	100	99.69	99.69	99.69	99.69
PPV_normal (%)	100	99.08	99.09	99.08	99.08
NPV_normal (%)	100	99.69	100	99.69	99.69
Acc_mild (%)	98.17	98.63	98.63	99.08	98.85
Se_mild (%)	95.95	100	96.96	100	97.97
Sp_mild (%)	98.82	98.23	99.11	98.82	99.11
PPV_mild (%)	95.95	94.28	96.96	96.11	97
NPV_mild (%)	98.82	100	99.11	100	99.40

Acc: Accuracy; Se: Sensitivity; Sp: Specificity; PPV: Positive Predictive Value; NPV: Negative Predictive Value.

**Table 5:** The results of the proposed methods used to classify COVID-19 patients.

	Random Forests	XGBoost	Ada Boost	AI-Score
Acc_severe (%)	96.84	98.94	91.57	98.94
Se_severe (%)	91.42	97.14	77.14	97.14
Sp_severe (%)	100	100	100	100
PPV_severe (%)	100	100	100	100
NPV_severe (%)	95.23	98.36	88.23	98.36
Acc_moderate (%)	97.89	97.89	90.52	98.94
Se_moderate (%)	93.33	93.33	100	96.66
Sp_moderate (%)	100	100	86.15	100
PPV_moderate (%)	100	100	76.92	100
NPV_moderate (%)	97.01	97.01	100	98.48
Acc_mild (%)	94.73	96.84	98.94	97.89
Se_mild (%)	100	100	96.66	100
Sp_mild (%)	92.30	95.38	100	96.92
PPV_mild (%)	85.71	90.90	100	93.75
NPV_mild (%)	100	100	98.48	100

Acc: Accuracy; Se: Sensitivity; Sp: Specificity; PPV: Positive Predictive Value; NPV: Negative Predictive Value.

## Data Availability

The code and models will be available upon request to authors and under restrictions regarding ethical aspects.
